# Vertical and In-Plane Electronic Transport of Graphene Nanoribbon/Nanotube Heterostructures

**DOI:** 10.3390/nano12193475

**Published:** 2022-10-04

**Authors:** Antonio Bernardo Felix, Monica Pacheco, Pedro Orellana, Andrea Latgé

**Affiliations:** 1 Physics Institute, Federal Fluminense University, Av. Litorânea, Niterói 24210-356, RJ, Brazil; 2 Physics Departament, Santa Maria University, Av. Espana, Valparaíso 2390123, Chile

**Keywords:** graphene nanostructures, transport, midgap states

## Abstract

All-carbon systems have proven to present interesting transport properties and are often used in electronic devices. Motivated by recent resonant responses measured on graphene/fullerene junction, we propose coupled nanoribbons/carbon-nanotube heterostructures for use as charge filters and to allow tuned transport. These hybrid systems are engineered as a four-terminal device, and we explore multiple combinations of source and collector leads. The armchair-edge configuration results in midgap states when the transport is carried through top/bottom terminals. Such states are robust against the lack of perfect order on the tube and are revealed as sharp steps in the characteristic current curves when a bias potential is turned on. The zigzag-edge systems exhibit differential negative resistance, with features determined by the details of the hybrid structures.

## 1. Introduction

Molecular transport has been addressed recently as a valuable possibility for driving electrons in convenient device architectures [1,2,3]. Furthermore, the advances in bottom-up growth processes of carbon-based materials have provided interesting scenarios to explore the possibility of accurate transport tuning [4,5,6,7]. Tunable carbon electronics based on hybrid junctions composed of graphene layers and fullerene molecules have recently been considered as a promising system with controllable resonance responses [8]. Moreover, on-surface chemistry techniques have provided a robust mechanism to synthesize quasi-free monolayer graphene and graphene nanoribbons (GNR), among other carbon-based systems, helping to solve various nanotechnological challenges [9,10]. The growth of five-atom-wide armchair nanoribbon was recently reported starting from precursor molecules [11], indicating a successful route for control-confined nanostructured systems. Following the partial unzipping process of multi-walled carbon nanotubes (CNTs), it is possible to fabricate cross-linked architectures of CNT/GNR hybrids [12], making soft films with enhanced electronic charge transfer.

Complex nanostructured materials have been proposed based on the coupling of graphene nanoribbons and other carbon allotropes [13,14,15,16]. The progress in inter-facial engineering and structural integration of carbon nanomaterials has led to a fruitful scenario for advanced electronic applications [17,18,19]. Anisotropic electrically conductive films based on highly aligned polyimide fibers containing hybrid materials of graphene nanoribbons and carbon nanotubes have been reported [12], providing for an open way of developing anisotropic conductive carbon-based nanomaterials. Numerical simulations describing the atomic configuration of hybrid films composed of graphene layers and single-walled carbon nanotubes have revealed the emergence of structural regions with increased density [20], corresponding to the data from experiments on synthesizing graphene/CNT films [21].

Previous theoretical works describing finite carbon nanotubes on a nanoribbon of the same width revealed Fano antiresonances and tuning conductance [22] when varying the topology of the hybrid systems. The zigzag arrangement has proven to exhibit robust and controllable negative differential resistance (NDR) at a given range of voltage values. Recent studies have reported NDR formation in randomly oriented graphene-like nanostructures up to 37 K and high on-current density [23]. Density functional studies have predicted that the electronic properties of graphene nanoribbon/nanotube systems are dependent on the atomic details of the hybrid structure and on the spin orientation [24].

Here, we adopt the scattering formalism to obtain transport transmission coefficients, assuming perfect matching between two nanoribbons (top/bottom) and a carbon nanotube inside them. Both zigzag and armchair edge geometries are considered. We follow a tight-binding approximation to describe the device. A multiple-mode approach is used in the case of armchair systems to analytically calculate the transmission function for the hybrid system. Standard real-space renormalization techniques are employed to obtain the system’s Green functions and electronic properties. Both results coincide perfectly well. The conductance for a finite CNT embedded into two graphene nanoribbons presents different behavior depending on the type of transport through the heterostructure. For in-plane transport, the conductance of the systems with armchair and zigzag graphene nanoribbons (AGNR and ZGNR) is similar to the case studied in our previous work [22]. The conductance of the hybrid AGNR/CNT/AGNR exhibited a series of Fano antiresonances due to the quantum interference effect between the continuum AGNR states and the CNT discrete states. On the other hand, the top/bottom (bottom/top) transport displays a series of resonances in the conductance typical of a resonant-tunneling device. All the recent experimental works in the synthesis of carbon-based materials with atomic precision mentioned above make us confident in the successful realization of the proposed heterostructure.

## 2. Tight-Binding Model

**Figure 1:**
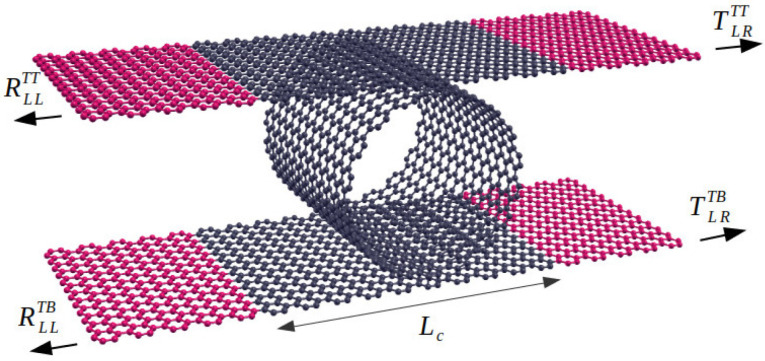
Schematic view of the prototype device discussed here: two N-armchair nanoribbons (top and bottom) with a finite (n,n)-CNT laying in between. The tube is perfectly superposed with ribbons. Gray colors are used to denote the central region (length $L_{c}$) and red to define the right/left, top/bottom leads.

The system is separated into two major parts, depicted in Figure 1. The central conductor system is composed of a finite carbon nanotube, and small top and bottom graphene nanoribbon slices (gray atoms in the figure) work as a scattering region. We use a π-band tight-binding Hamiltonian, written as

(1) $$H=H_{T}^{R,L}+H_{B}^{R,L}+H_{C}+H_{coupl}$$

where $H_{T(B)}^{R(L)}$ corresponds to the right (left) and top (bottom) lead, considered as pristine nanoribbons, perfectly matching the central part $H_{C}$. The system is not passivated with hydrogen atoms.

The central hybrid system and the leads are connected via a coupling Hamiltonian, $H_{coupl}$. The leads and central Hamiltonians are defined in terms of on-site and hopping energies using standard tight-binding models for graphene materials, i.e., $H=\sum_{ij}t_{ij}c_{i}^{†}c_{j}$, that have been successfully adopted to describe different all-carbon systems. Following this scheme, the on-site energy $t_{ii}=\epsilon_{0}$ is taken as the zero energy, and the hopping energy $t_{ij}$ between carbon atoms in the nanoribbon and within the finite CNT is considered equal to 2.75 eV [25]. A smaller value is chosen for hopping between the nanoribbon and tube atoms at the top and bottom junctions. The presence of gases and strain may alter the value of the coupling energy between the distinct parts of the hybrid structure. Here, we have used a standard value [22], $t^{\prime }=0.2t$. For both armchair and zigzag cases, the coupling between the tube and the ribbons involves A–B dimer pairs for the two carbon systems, A and B denoting the two graphene sublattices. Actually, other models for this hopping parameter may be considered, taking into account, for instance, a cutoff distance between the tube and nanoribbon neighbour atoms. However, the main transport results we want to highlight here are robust against such model details.

Standard numerical procedures based on real-space renormalization techniques are adopted to obtain the Green functions of the system recursively [26,27,28,29,30] and to calculate the total and local electronic density of states of the all-carbon molecular junction. Transport properties are explored by calculating electronic conductance and currents within the Landauer formalism [31]. Within this context, the central advanced and retarded Green function is provided by

(2) $$G_{C}^{a,r}\left(E\right)={\left[\omega -H_{C}-\Sigma_{LT}^{a,r}-\Sigma_{RT}^{a,r}-\Sigma_{LB}^{a,r}-\Sigma_{RB}^{a,r}\right]}^{-1}\;,$$

with ω=E±iη, η being an infinitesimal number and $\Sigma_{LR/TB}^{a,r}$ corresponding to left and right/top and bottom self-energies, provided by the corresponding surface Green functions, from which the coupling matrices are obtained:

(3) $$\Gamma^{LR/TB}\left(E\right)=i\left(\Sigma_{LR/TB}^{r}\left(E\right)-\Sigma_{LR/TB}^{a}\left(E\right)\right)\;\;.$$

Finally, to perform the calculation of the electronic conductance from the top left to bottom right leads, we use the energy-dependent transmission provided by

(4) $$T(E)=\mathrm{Tr}\left[\Gamma^{L/T}G_{C}^{r}\Gamma^{R/B}G_{C}^{a}\right]\;\;.$$

Considering two leads at different chemical potentials μ=±V/2 and a linear potential drop within the scattering region, the current at zero temperature is provided by $I=\frac{2e}{h}\int_{-V/2}^{V/2}T\left(E\right)dE$. The tube is considered at null potential energy for simplicity.

Alternatively, in the case of armchair nanostructures, we adopt multiple-mode approximation [32] for the complex system composed of top and bottom armchair nanoribbons and the commensurable armchair tube localized between the ribbons to derive the transmission coefficient. For a single armchair graphene nanoribbon, the equation of motion for the probability amplitudes of finding an electron at the j,m site of the atomic *A* and *B* sublattices is provided by

(5) $$\begin{array}{c} \left(\epsilon -\epsilon_{p}\right)\Psi_{jm}^{A,p}=-t\Psi_{jm}^{B,p}-t\Psi_{j-1,m+1}^{B,p}-t\Psi_{j-1,m-1}^{B,p}\;\\ \left(\epsilon -\epsilon_{p}\right)\Psi_{jm}^{B,p}=-t\Psi_{jm}^{A,p}-t\Psi_{j+1,m-1}^{A,p}-t\Psi_{j+1,m+1}^{A,p}\;\;, \end{array}$$

with *t* being the same hopping energy mentioned above and *p*=top and bottom. Taking into account the translation symmetry along the isolated ribbons, the electronic wave function may be written as $\Psi_{j,m}^{A(B),p}=\Phi_{j}^{A(B),p}e^{iqm}$, with *q* being defined by the lateral ribbon confinement [33], and $\Phi_{j}^{A(B),p}$ the wavefunction amplitude of site *j* in the A(B) nanoribbon sublattice. Using the above and considering a coupled finite carbon nanotube (m,m) between the ribbons of the same extension and chirality of the armchair GNRs, we obtain

(6) $$\left(\epsilon -\epsilon_{p}\right)\Phi_{j}^{A(B),p}=-t\Phi_{j}^{B(A),p}-\gamma \Phi_{j-1}^{B(A),p}-t^{\prime }\alpha_{l}\left(\beta_{l}\right)f_{l,j}\;\;,\;\;\;$$

with γ=2tcosq, $\alpha_{r}$, and $\beta_{r}$ denoting the electronic wave functions of the trapped CNT, and with $f_{l,j}=1$ for $f_{1,j}$ and $f_{M/2+1,j}$ at the top and bottom GNRs, respectively, where *j* are the junction sites at the top and bottom ribbons. For all other combinations, $f_{l,j}=0$. Notice that the number of dimers along the tube circumference is provided by *M*, corresponding to a (M/2,M/2) tube.

Equivalent equations can be written for the tube:

(7) $$\begin{array}{c} \left(\epsilon -\epsilon_{T}\right)\alpha_{l}=-t\beta_{l}-\gamma \;\beta_{l-1}-t^{\prime }\Phi_{j}^{A,p}f_{l,j}\; \end{array}$$

(8) $$\begin{array}{c} \left(\epsilon -\epsilon_{T}\right)\beta_{l}=-t\alpha_{l}-\gamma \;\alpha_{l+1}-t^{\prime }\Phi_{j}^{B,p}f_{l,j}\;. \end{array}$$

Applying the boundary conditions for the tube atom positions closest to the up and bottom nanoribbons, l=1 and l=M/2+1, we obtain a set of eight analytical equations that provide us with the transmission and reflection coefficients for the different transport channels involving top and bottom leads at the right and left positions. Here, we concentrate on the left to right electronic transport through a single ribbon, top/top (T/T) or bottom/bottom (B/B), and investigate the top/bottom (T/B) transport mediated by the CNT molecule. The numerical results obtained for the transmission coefficients relative to the armchair hybrid systems using the Green function formalism are in total agreement with the semi-analytic predictions discussed above.

## 3. Results

**Figure 2:**
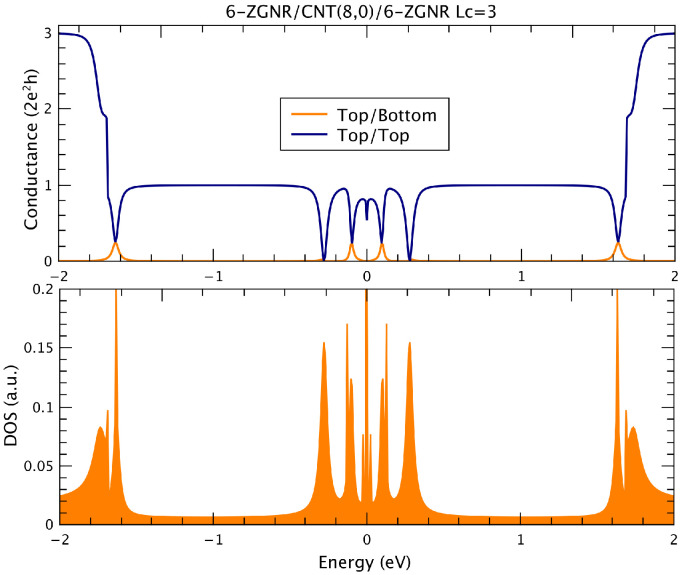
T/T (dark blue curve) and T/B (orange curve) conductance results (**top panel**) and DOS (**bottom panel**) for a 6-ZGNR/CNT(8,0)/6-ZGNR system.

In the following, we discuss the transport properties of such nanoscaled devices, considering both zigzag and armchair geometries. We focus first on the zigzag-edged systems. Figure 2 shows the conductance results for electrons coming from the left to the right top leads (blue curve) and to the right bottom terminal (orange curve) in a 6-ZGNR/CNT(8,0)/6-ZGNR structure. In the bottom part, we present the corresponding density of states of such devices, essentially illustrating the energy range related to the first transport channel and the transition to the second one. Typical van Hove singularities are exhibited as a consequence of the quasi-one-dimensional nature of the isolated components. It is interesting to note the presence of non-null top to bottom (T/B) transport exactly when sharp depression on the top to top (T/T) conductance is verified, revealing the link between both conductance and the discrete tube states.

**Figure 3:**
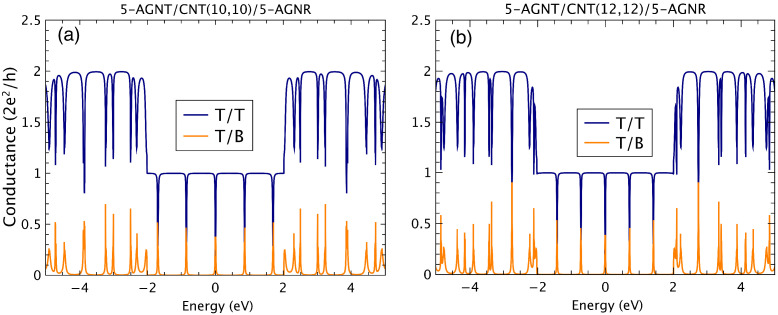
(Color online) Conductance results for the 5-AGNR/(n,n) CNT/5-AGNR, with *n* = 10 (**a**) and 12 (**b**) for T/T and T/B transport.

Results for the conductance of metallic armchair hybrid systems, composed of 5-AGNR/CNT(n,n)/5-AGNR, are shown in Figure 3 for T/T (blue curves) and T/B (orange curves) transport, corresponding to tubes given by *n* = 10 and 12. Similarly to the zigzag systems, there is an important correspondence between the conductance depressions occurring in the top to top movement at the energies where localized states within the conductance gap are manifested in the top to bottom transport.

**Figure 4:**
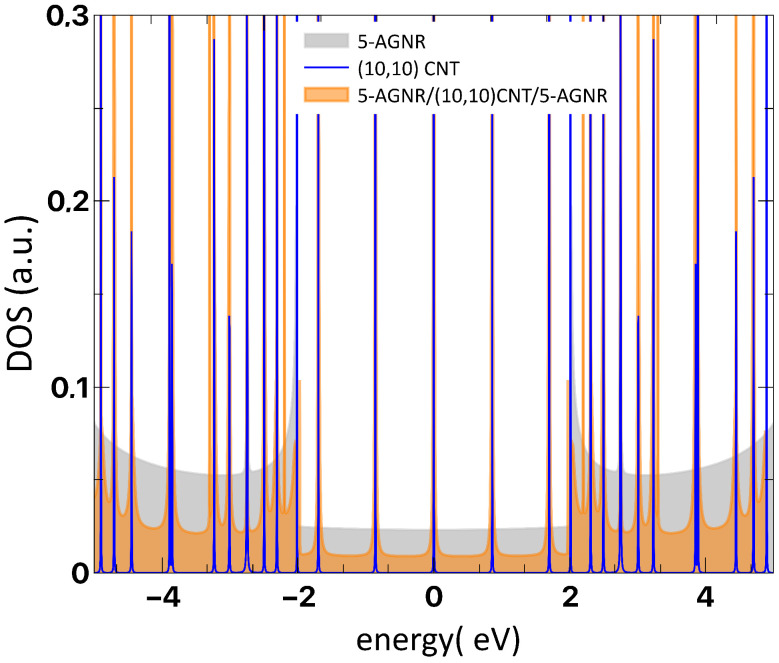
(Color online) DOS results for a 5-AGNR/(10,10)CNT/5-AGNR system, with *n* = 10 (shaded orange), for a single 5-AGNR (shaded gray), and for the finite isolated (10,10) CNT (blue lines).

To highlight the origin of the antiresonances and resonances shown in Figure 3, we present in Figure 4 the energy positions of the finite CNT states (blue lines) superposed to the density of states (DOS) of the infinite graphene nanoribbon 5-AGNR (orange shaded) and the hybrid system (gray shaded). We clearly observe the agreement between the antiresonance energy positions of the conductance results shown in Figure 3a and the bound states of the finite (10,10) CNT. In the case of the metallic armchair-edged nanostructures, the electronic tunneling through the carbon tube is revealed by such “midgap states”, which are very particular to the metallic family. In fact, these features are absent when considering semiconducting armchair-edged geometries for Top/Bottom transport; i.e., within the semiconducting gap of the related hybrid system, no resonant states appear. Differently from the zigzag geometry, the emergence of such special localized states allows for a quite different characteristic curve (current versus bias voltage) driven by the sequence of the delta-like states.

**Figure 5:**
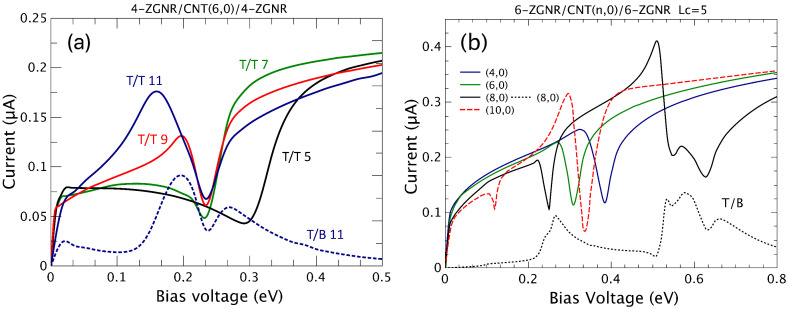
(Color online) Current (T/T) versus voltage potential for a (**a**) 4-ZGNR/(6,0)CNT/4-ZGNR system with $L_{c}$ = 5, 7, 9, and 11 and (**b**) 6-ZGNR/(n,0)CNT/6-ZGNR and fixed $L_{c}$ = 5 for different CNT radii (*n* = 4, 6, 8, and 10). T/B currents are shown in the lower part of both figures for comparison.

In the following, we address current versus bias calculations for both zigzag and armchair configurations when the leads are submitted to a finite bias potential. First, we consider the zigzag-edged hybrid systems. Figure 5a shows T/T (continuous curves) and T/B (dashed lines) results of the current for 4-ZGNR/CNT(6,0)/4-ZGNR molecular devices taking into account different conductor lengths $L_{c}=$ 5, 7, 9, and 11.

Similarly to what was previously found in the case of a hybrid system composed of a nanotube on a single nanoribbon, negative differential resistances (NDRs) are verified at particular ranges of bias voltage. Depicted in the figure is an example of top/bottom current for $L_{c}=11$ (dashed blue curve). Although the current signal corresponding to transport through the carbon tube from the top and bottom nanoribbons is lower than the top to top channel, extra zones of NDRs are obtained, offering new application possibilities. The general current profiles depend on the length of the center conductor spacer, as it defines the extent to which the potential drop occurs.

We explored the dependence of the current dip as a function of the bias voltage for different tube radii, fixing the nanoribbon width as 6-ZGNR and the size of the central conductor as $L_{c}=5$. The results shown in Figure 5b exhibit the same main features, revealing a dislocation of the peak/dip to lower bias values as the tube radius increases. In fact, the dips in the current can be modeled by a Fano-line shape [34,35], as

(9) $$F\left(\varepsilon \right)=\gamma \frac{{(\varepsilon +q)}^{2}}{1+\varepsilon^{2}}$$

where γ is a proportionality constant, $\varepsilon =(V-V_{r})/\Gamma$, with $V_{r}$ and Γ being the Fano antiresonance position and width, respectively, and *q* is a complex number with real and imaginary parts. Here, the Fano antiresonances appear due to the quantum interference between the continuum states in the graphene nanoribbons and the discrete states in the CNT.

As expected, the negative differential resistance is evidenced in different bias potential ranges depending on the details of the nanostructured systems (tube radius, conductor length, GNR width) and on the local temperature [22]. As the bias voltage increases, extra resonant states are addressed, revealing additional features in the current, as is highlighted for the cases of (8,0) and (10,0) tubes.

**Figure 6:**
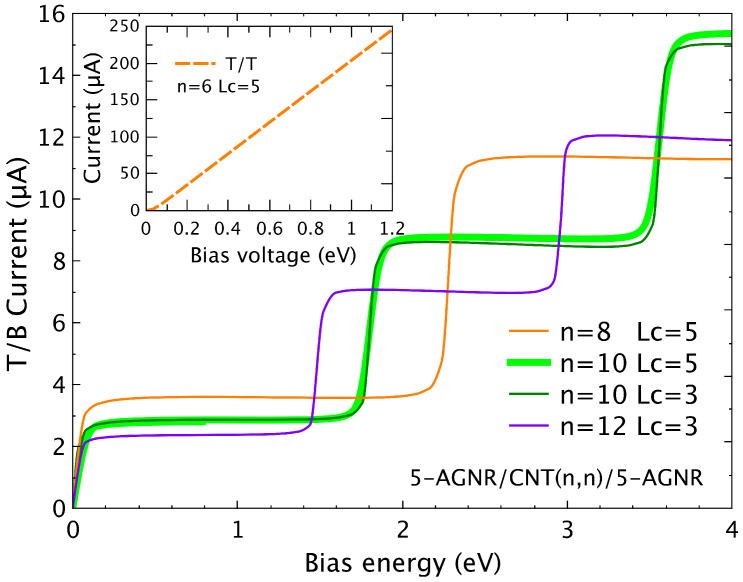
(Color online) Current versus bias potential applied on the left top and right bottom leads for a 5-AGNR/CNT(n,n)/5-AGNR for *n* = 8, 10, and 12, and conductor spacer $L_{c}$ = 3 and 5. Inset: T/T Current for *n* = 6 and *$L_{c}$* = 5.

Results for the current when the leads are submitted to a finite bias are shown here for the AGNR/CNT/AGNR devices for a (3p + 2) metallic family (with p being an integer). While the top to top current presents the same ohmic-like behavior due to the central plateau found for low energies, the top to bottom results exhibit a sequence of steps at bias voltages $V_{R}=2\epsilon_{R}$, these being the resonance energies. In Figure 6, we illustrate such a step hallmark found for the hybrid system 5-AGNR/CTN(n,n)/5-AGNR considering different combinations of tube radii and conductor spacers. As expected, the step change is essentially defined by the tube radii, as is confirmed for the cases with *n* = 10 and two different conductor spacers, $L_{c}=$ 3 and 5 (green curves).

In fact, for the top/bottom transport corresponding to the first miniband, the transmission can be written as a sum of Breit–Wigner line shapes around the resonant energies $\varepsilon_{j}$ with widths $\Gamma_{j}$. Then,

(10) $$T\left(\varepsilon \right)=\sum_{j}\frac{\Gamma_{j}^{2}}{{\left(\varepsilon -\varepsilon_{j}\right)}^{2}+\Gamma_{j}^{2}}\;\;.$$

By integrating over the energy range [−V/2,V/2], we obtain an approximated expression for the current at zero temperature:

(11) $$I=\frac{2e}{h}\sum_{j}\Gamma_{j}[\arctan\left(\frac{V-2\epsilon_{j}}{2\Gamma_{j}}\right)+\arctan\left(\frac{V+2\epsilon_{j}}{2\Gamma_{j}}\right)]\;.$$

From the above equation, we can conclude that in the vertical transport through the carbon molecule, the transmission channels are provided by the eigenvalues of the finite armchair CNT and do not depend on the central conductor spacer provided by the $L_{c}$ value.

To emphasize the difference between the T/T and T/B transport features, we plot in the inset of Figure 6 the current related to transport through the top nanoribbon (T/T) for a (6,0) CNT and considering $L_{c}=5$. The revealed ohmic behaviour is similar to the results previously predicted for the metallic armchair hybrid devices considering a single nanoribbon [22], and is justified by the existence of a large plateau of complete transmission for the low energy range.

Taking into account the fact that defects and disorder are in general unavoidable in practical materials, a further investigation of the transport effects due to breaking the perfect symmetry of the discussed armchair hybrid devices was performed as well. Isolated impurities or a set of substitutional impurities were considered through the nanotube walls by assuming different on-site energy values. In addition to a discrete lack of electron-hole symmetry and quite small energy shifts in the conductance results, this type of disorder does not reveal important changes in the step-like features of the current for such hybrid systems.

## 4. Final Remarks

In this study, we have addressed the electronic transport properties of an all-carbon structure composed of a CNT molecule sandwiched between two graphene nanoribbons. We have found that the T/T (or B/B) transport response for zigzag-edged junctions exhibits similar general behaviors to those found when the tube is on a single nanoribbon. The T/T (or B/B) transport reveals robust negative differential resistance at a given range of voltage values that may be controlled, as previously verified for simpler hybrid systems. The tube radii, nanoribbon width, and conductor spacers provide characteristic negative differential resistance details. The NDR is found in the T/B current in the zigzag geometry. In the case of metallic armchair nanoribbons, the transport from top to bottom (and vice versa) through the carbon molecule was proven to exhibit interesting features, such as the formation of midgap states, typical of a resonant-tunneling device. The positions of the conductance resonances for the vertical transport correspond to the CNT eigenenergies. The related characteristic I/V curves are marked by a sequence of plateaus occurring at the half value of the emergent resonant energies in the gap. Our findings indicate that the main responses are robust against disorder in the nanotube walls. The obtained fine-tuning of electronic transmission through the studied hybrid heterostructure allows us to predict interesting routes of tunneling transport, taking into account such all-carbon nanodevices.

## Data Availability

The data presented in this study are available on request from the corresponding author.
